# DXA beyond bone mineral density and the REMS technique: new insights for current radiologists practice

**DOI:** 10.1007/s11547-024-01843-6

**Published:** 2024-07-30

**Authors:** Carmelo Messina, Stefano Fusco, Silvia Gazzotti, Domenico Albano, Gloria Bonaccorsi, Giuseppe Guglielmi, Alberto Bazzocchi

**Affiliations:** 1 IRCCS Istituto Ortopedico Galeazzi, 20161 Milan, Italy; 2 Department of Biomedical Sciences for Health, University of Milan, 20133 Milan, Italy; 3 Postgraduate School of Diagnostic and Interventional Radiology, University of Milan, 20122 Milan, Italy; 4 Diagnostic and Interventional Radiology, IRCCS Istituto Ortopedico Rizzoli, 40136 Bologna, Italy; 5 Department of Biomedical, Surgical and Dental Sciences, University of Milan, 20122 Milan, Italy; 6 Department of Translational Medicine, Menopause and Osteoporosis Center, University of Ferrara, Ferrara, Italy; 7 Radiology Unit, Department of Clinical and Experimental Medicine, Foggia University School of Medicine, 71122 Foggia, Italy; 8 Radiology Unit “Mons. Dimiccoli” Teaching Hospital, Barletta (BT), Italy; 9 Radiology Unit, Scientific Institute “Casa Sollievo Della Sofferenza” Hospital, 71013 San Giovanni Rotondo, Italy

**Keywords:** Dual-energy X-ray absorptiometry (DXA), Bone mineral density (BMD), Trabecular Bone Score (TBS), Bone Strain Index (BS), Radiofrequency Echographic Multi-Spectrometry (REMS), Osteoporosis

## Abstract

Osteoporosis is the most prevalent skeletal disorder, a condition that is associated with significant social and healthcare burden. In the elderly, osteoporosis is commonly associated with sarcopenia, further increasing the risk of fracture. Several imaging techniques are available for a non-invasive evaluation of osteoporosis and sarcopenia. This review focuses on dual-energy X-ray absorptiometry (DXA), as this technique offers the possibility to evaluate bone mineral density and body composition parameters with good precision and accuracy. DXA is also able to evaluate the amount of aortic calcification for cardiovascular risk estimation. Additionally, new DXA-based parameters have been developed in recent years to further refine fracture risk estimation, such as the Trabecular Bone Score and the Bone Strain Index. Finally, we describe the recent advances of a newly developed ultrasound-based technology known as Radiofrequency Echographic Multi-Spectrometry, which represent the latest non-ionizing approach for osteoporosis evaluation at central sites.

## Introduction

Osteoporosis is the most prevalent skeletal disorder, a condition that is associated with significant social and healthcare burden. This disease is defined by a combination of reduced bone mass and microarchitecture deterioration, leading to increased risk of fragility fracture typically occurring at the spine, femur, and distal radius. In the elderly, osteoporosis is commonly associated with sarcopenia, which represents a progressive and age-related loss of muscle mass; the combination of both diseases is now recognized as osteosarcopenia, further increasing the risk of fracture.

Several imaging techniques are available for a non-invasive evaluation of bone and body composition to diagnose osteoporosis and sarcopenia. This review specifically focuses on dual-energy X-ray absorptiometry (DXA), as this technique offers the possibility to evaluate bone mineral density (BMD) and body composition parameters with good precision and accuracy. DXA is also able, in the lateral acquisition, to estimate the amount of aortic calcification for cardiovascular risk estimation. Additionally, new DXA-based parameters have been developed in recent years to further refine fracture risk estimation, such as the Trabecular Bone Score (TBS) and the Bone Strain Index (BSI). This article is aimed at revising the current evidence available for the use of DXA in this scenario, providing radiologists with up-to-date information on newer diagnostic possibilities offered by this technique.

This article also presents a newly developed ultrasound-based technology known as Radiofrequency Echographic Multi-Spectrometry (REMS), which represents the latest non-ionizing approach for osteoporosis evaluation at central sites. REMS technology is able to analyze the raw unfiltered ultrasound signal acquired at lumbar spine and proximal femur, obtaining BMD ultrasound values that showed to be very accurate compared to DXA.

## DXA-based Trabecular Bone Score

BMD assessed by DXA is the main criterion for the diagnosis of osteoporosis and for the prediction of fragility fracture risk: BMD measures bone quantity and is therefore strongly related to bone resistance [1].

However, many subjects with fragility fractures show slightly low or even normal BMD values. This is because other skeletal features such as bone microarchitecture, bone remodeling, and bone geometry contribute to bone quality and influence fracture risk [2].

To better assess bone quality, other imaging techniques have been employed, such as quantitative computed tomography (QCT), high-resolution peripheral QCT (HRpQCT), and high-resolution magnetic resonance imaging (MRI). However, these technologies have higher costs, lower availability, and higher ionizing radiation dose (for QCT) compared to DXA [3]. Therefore, alternative DXA-based parameters have been developed over time to refine fracture risk prediction and investigate skeletal features other than BMD.

The Trabecular Bone Score (TBS), introduced in early 2010, is a DXA-based textural index that evaluates gray-level variations of each pixel of a DXA image: These variations depend on the specific X-ray absorption properties of the corresponding 3D tissue, thus reflecting the tissue microarchitecture. In this way, TBS can provide a valid indirect estimation of lumbar trabecular microarchitecture, even if a direct measurement is not feasible (due to technical limitations related to pixel width of DXA image, which is about four time larger the mean size of trabeculae) [4]. TBS is calculated by a specific software (TBS iNsight; Medimaps, Plan-les-Ouates, Switzerland) on the lumbar spine DXA image, in the same region of interest used for BMD measurement [5]. High TBS values correspond to a dense trabecular structure and thus a good microarchitecture; on the contrary, a degraded bone microarchitecture matches low TBS values [6].

**Fig. 1:**
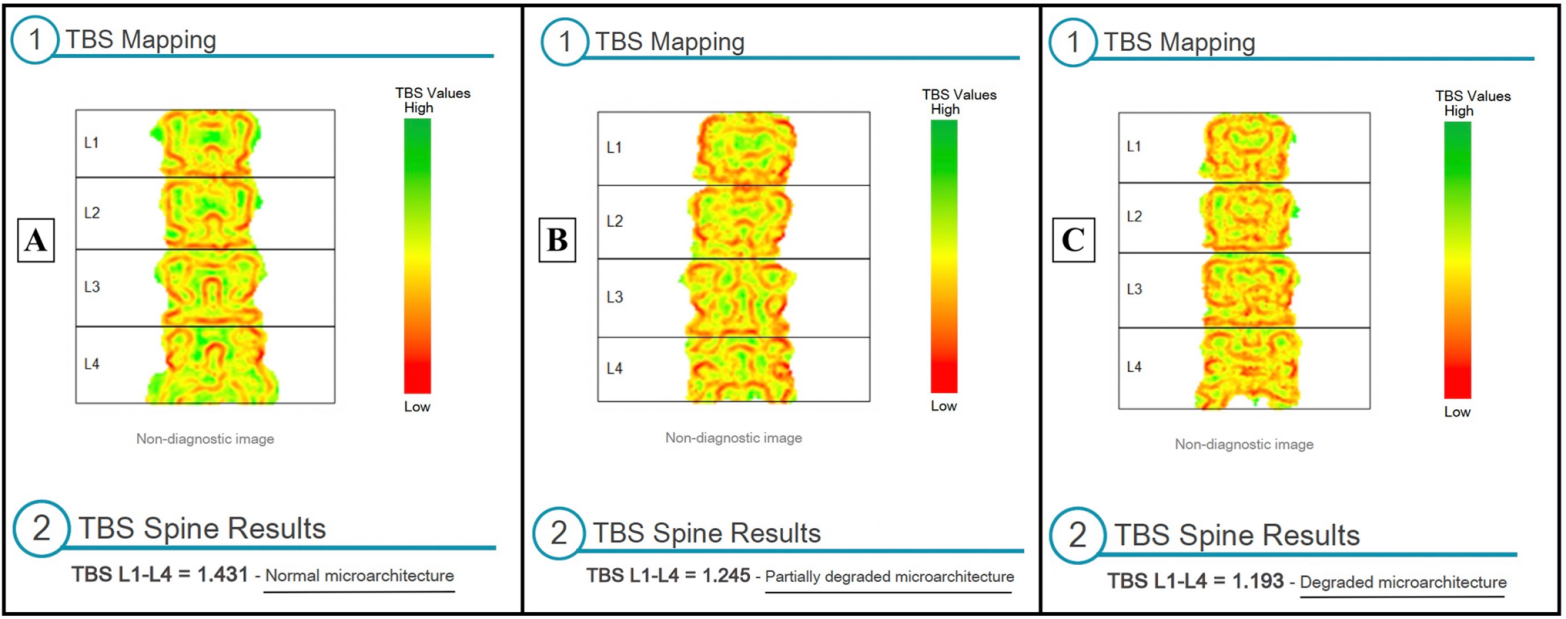
Lumbar spine TBS from three different subjects presenting with normal, partially degraded, and degraded microarchitecture. Of note, a color map is displayed over the lumbar spine region of interest: Green is associated with higher TBS values, while red is associated with degraded microarchitecture

A large meta-analysis developed threshold values: TBS > 1.310 is considered normal and corresponds to a normal bone microarchitecture and a low fracture risk; TBS values between 1.23–1-31 identify a partially degraded microarchitecture; TBS < 1.230 corresponds to degraded microarchitecture and a higher risk of fractures, in both men and women [7]. An example of three different TBS reports showing normal, partially degraded, and degraded microarchitecture is shown in Fig. 1.

In 2015, the International Society for Clinical Densitometry (ISCD) TBS task force published a position paper to develop official positions on the incorporation of TBS in clinical practice [5]. The paper highlights that low TBS values are associated with an increase in both prevalent and incident fragility fractures. In particular, the use of TBS is recommended for the assessment of vertebral, hip, and major osteoporotic fracture risk in postmenopausal women and for hip fracture risk in men older than 50 years [5]. TBS also demonstrated to predict fracture risk independently of DXA-BMD and clinical risk factors. Therefore, TBS was incorporated into the FRAX® tool to adjust 10-year fracture probability [5, 7].

A limitation of TBS is that its application is currently not advised for monitoring patients under antiosteoporotic treatment [8]. This is explained by the fact that TBS has a slightly lower precision compared to BMD, leading to higher least significant values (LSC) and consequently longer interval to detect changes over time [5, 9].

A technical issue which has characterized TBS software from the beginning was the underestimation of TBS value due to the individual’s regional soft tissue thickness. Consequently, TBS showed a negative correlation with BMI, as opposed to BMD and biomechanical properties of the bone. An updated version of TBS algorithm (TBSv4.0) uses a compensation for the thickness of the lumbar soft tissue to overcome this issue and demonstrated a positive correlation of TBS values with BMI [10].

Regarding new applications, a beta version of TBS software was recently developed for the evaluation of the hip (TBS-Hip—v1.0, Medimaps group, Geneva, Switzerland). A recent study conducted on women of 65 years or older who were residents of long-term care facilities tried to evaluate the performance of this new algorithm. The study showed a moderate correlation between BMD and TBS-Hip results at total hip, femoral neck, and greater trochanter, suggesting that TBS-Hip can potentially provide information on bone architecture of the hip, as already demonstrated for the spine [11].

## DXA-based Bone Strain Index

As above mentioned, TBS well predicts fracture risk by reflecting the microarchitecture of the bone. However, it is not able to evaluate bone strength or fatigue, two parameters that impact on the bone resistance to loads.

In this scenario, another DXA-derived index called Bone Strain Index (BSI) was introduced in 2019, with the aim of further exploring bone quality features. BSI provides information about bone strength and resistance to loads, which were not assessed from existing indices [12].

This index is based on the application to DXA images of a mathematical model defined as finite element analysis (FEA). FEA model relies on the principle that a complex object can be analyzed by transforming it into many simpler and smaller elements (i.e., “finite elements”) that can easily be managed to simulate any specific phenomenon [13].

The finite element analysis of the lumbar or femur DXA scan is automatically performed by BSI software (Tecnologie Avanzate s.r.l., Torino, Italy), starting from the division of the DXA area into small triangles, following the mapping contour of DXA image. Once the DXA image is divided in “finite elements” (the triangles), a FEA mathematical model is generated for both lumbar spine and proximal femur [14]. At the lumbar spine, the software simulates the gravitational force by applying a load to the upper vertebral plate, while a constraint is applied to the lower plate [15]. The magnitude of the load acting on the upper plate derives from a model which simulates forces in standing position, also depending on subject’s weight and height [16].

At the femur site, BSI algorithm tries to simulate a lateral fall, with a patient-specific impact force applied to the greater trochanter area and the constraints applied on the femoral head and the inferior part of the shaft [17].

The software generates a graphical representation of bone strain amount, allowing an easy identification of the areas characterized by higher strain concentration. Thus, the mean BSI value reflects the average bone equivalent strain, with higher BSI values corresponding to higher strain level and therefore to higher fracture risk. On the other hand, lower BSI values are indicative of a bone less subjected to strain, thereby at lower risk of fracture.

**Fig. 2:**
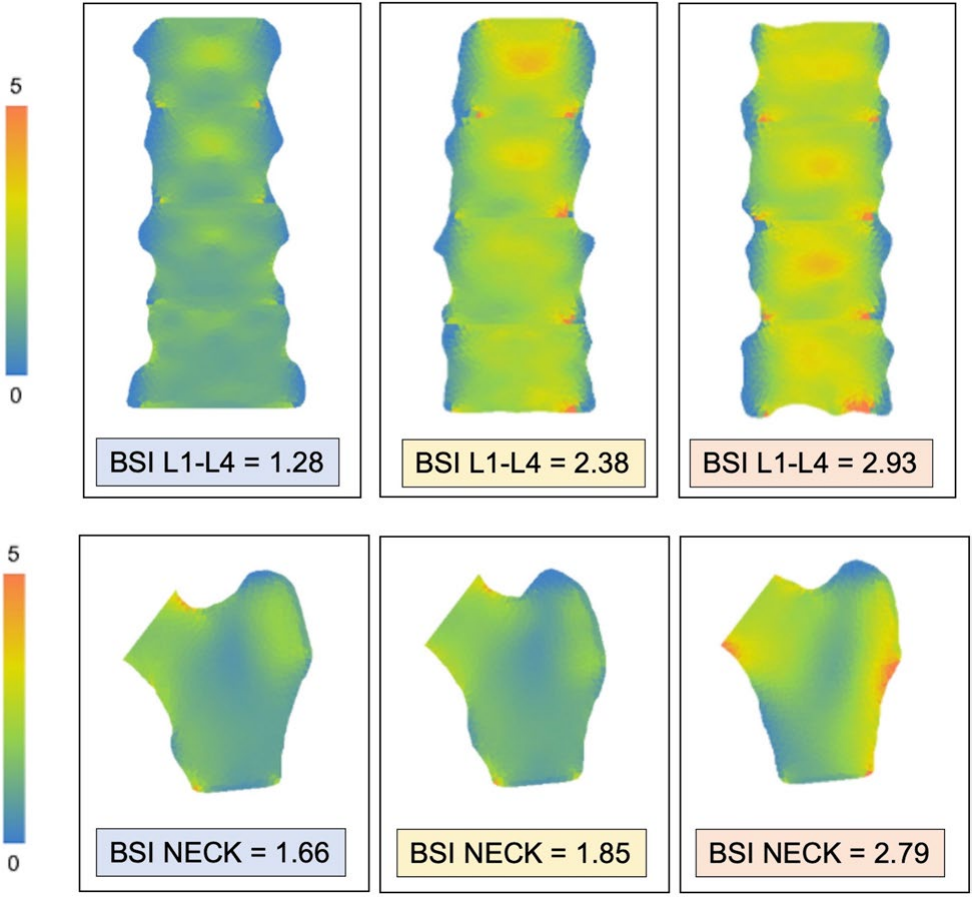
Examples of normal, low, and poor bone resistance to strain measured with BSI at lumbar spine (top) and proximal femur (bottom). Of note, the higher the BSI value (expressed as shades or orange and red), the higher is the strain level to bone, with increased fracture risk. For postmenopausal Italian women, the following thresholds have been proposed: BSI ≤ 1.7 = normality; BSI between 1.7 and 2.5 = low resistance to strain; BSI ≥ 2.5 = poor resistance to strain. [18]

BSI threshold values for the Caucasian population have been proposed in a study on postmenopausal Italian women: BSI ≤ 1.7 corresponds to normality; BSI between 1.7 and 2.5 reflects a low resistance to strain; BSI > 2.5 is related to poor resistance to strain [18]. Figure 2 shows some examples of different BSI diagnoses at lumbar spine and femoral neck.

Given its recent introduction, literature on BSI is rapidly growing and clinical studies have showed BSI usefulness in both primary and secondary osteoporosis. In osteoporotic patients, BSI values were found to be positively correlated with an increase of risk of fragility fractures at the lumbar spine and the hip in postmenopausal women, regardless of FRAX® [19]. Moreover, studies investigated the capability to predict the occurrence of fragility re-fracture, showing that BSI is an accurate predictive index of re-fracture [20, 21].

Regarding secondary osteoporosis, BSI can be useful in recognizing subjects at high risk for fragility fractures among patients with primary hyperparathyroidism [22, 23]. Lastly, BSI showed its usefulness even among young patients with neurofibromatosis type I (NF1), with higher BSI values in pubertal patients in comparison with prepubertal ones, implying a decrease in bone resistance to loads in older pediatric patients with NF1 [24].

In terms of monitoring antiosteoporotic treatment, few data are available in the literature. One recent study demonstrated that anabolic therapy with teriparatide seems to improve BSI, suggesting an increase of bone strength as a teriparatide effect [25].

## REMS

In recent years, a new ultrasound-based non-ionizing technique for the evaluation of BMD, bone fragility, and fracture risk has been introduced in clinical practice. This technique is called Radiofrequency Echographic Multi-Spectrometry (REMS) and is based on the spectral analysis of the whole raw unfiltered ultrasound signals (also called radiofrequency (RF) ultrasound signals) that are reflected from the bony surface. Conversely from traditional ultrasound techniques in bone densitometry, typically applied to peripheral sites (such as the calcaneus), REMS can be applied to central sites such as the lumbar spine and the femoral neck [26, 27].

**Fig. 3:**
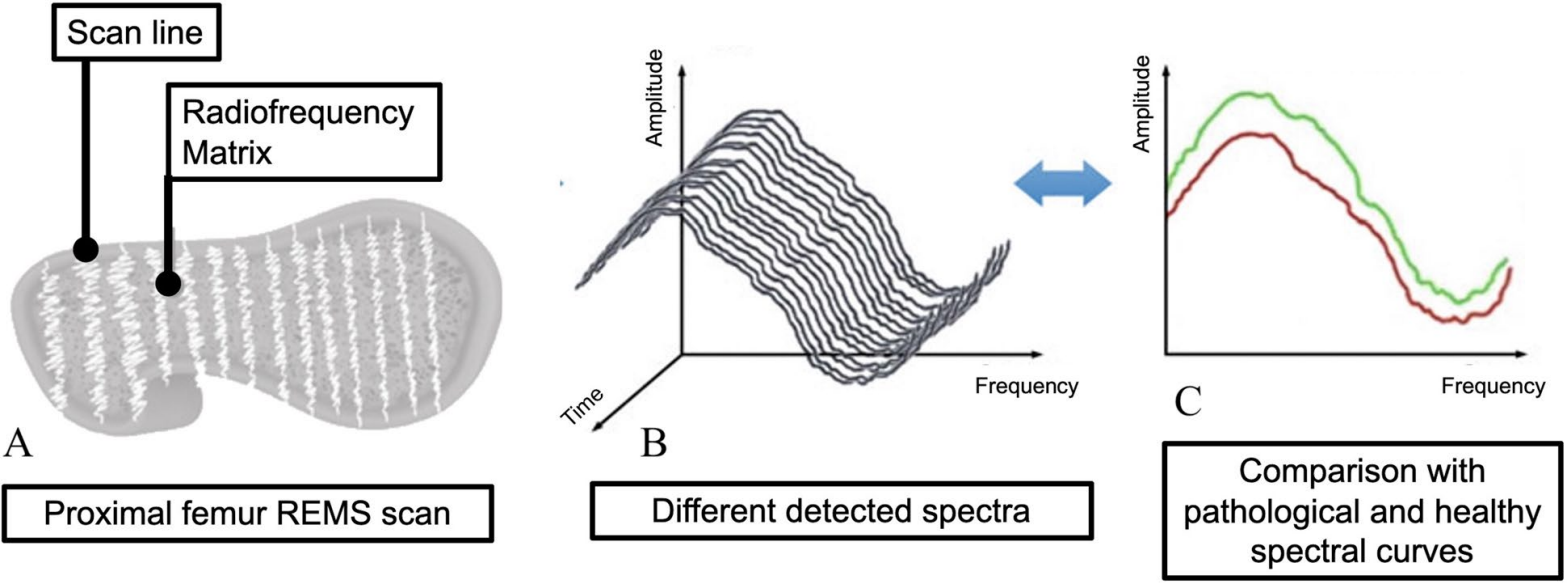
Example of working principle of REMS technology at proximal femur (image **a**), which is analogous to that at lumbar spine. Data from patient’s different spectra are analyzed (image **b**) and compared with reference models of healthy and pathologic spectral curves (image **c**). Such comparison allows for the generation of quantitative (BMD, T-score, and Z-score) and qualitative (Fragility Score) parameters

**Fig. 4:**
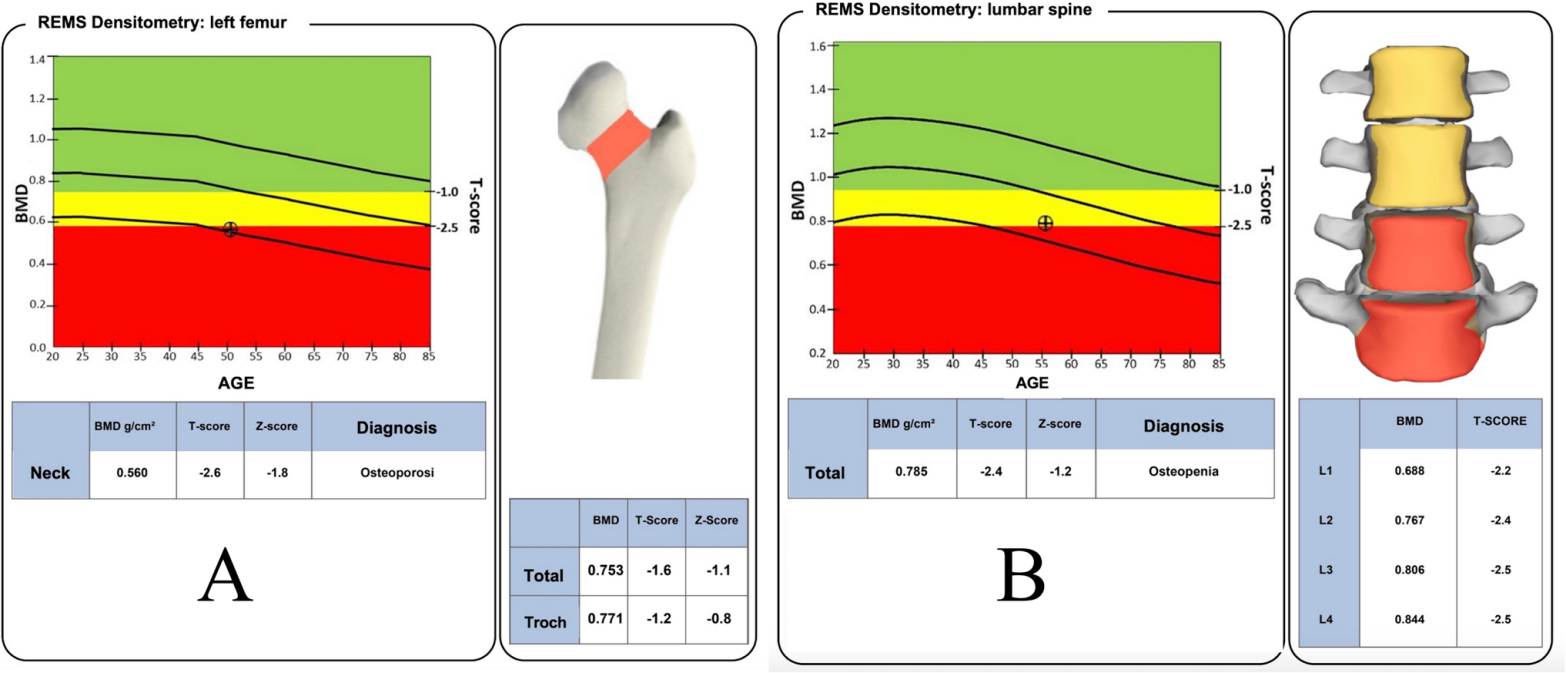
Example of a REMS report at proximal femur (**a**) and lumbar spine (**b**). BMD and T-score values are provided for femoral neck and total spine (L1–L4), but also at other regions of interest (total femur, trochanter, single vertebrae). The final diagnosis is done according to femoral neck and total spine T-score

What is new in the REMS technology is that the whole spectrum of information from the reflected “raw” unfiltered ultrasound wave is used to obtain information from bone, differently from conventional ultrasound devices, which use a limited part of the reflected wave to produce the B-mode image. REMS technology acquires and analyzes the whole RF, extracting a specific RF spectrum from the given sonographic bony layer, being able to identify and analyze the cortical and trabecular part of the bone [28]. More in detail, the REMS scan is acquired by placing the ultrasound probe on the specific anatomical site (abdomen for the lumbar spine or hip for the femoral neck). The operator is asked to adjust the scan depth in order to identify and properly visualize the vertebral or femoral cortical bone interface. After adjusting the transducer focus immediately above the cortical bone, REMS software is capable to automatically detect the region of interests (ROIs) at each site. See Fig. 3 for a schematic example of REMS acquisition working principle at femoral neck. The acquisition of the RF signal sets the basis for the following calculations that allow for the quantitative evaluation of BMD. For each sonographic line, a corresponding spectrum of radiofrequency is obtained, allowing at the same time to identify ROIs from trabecular bone and exclude bone signal artifacts (such as those originating from osteophytes) thanks to the software ability to identify abnormal spectral characteristics. A final trabecular patient-specific spectrum is generated, which is then automatically compared by the software to reference models for normal and osteoporotic bones, matched by gender, age, and BMI [29]. The whole procedure allows for the calculation of the percentage of analyzed spectra that were classified as “osteoporotic,” which is called the “osteoporotic score.” This score is therefore transformed by linear equations into ultrasound BMD values (US-BMD), which are expressed as g/cm^2^, and enables the generation of T-score and Z-score values by quantitative comparisons with the NHANES reference curves [26, 27]. A typical REMS report is provided in Fig. 4.

Several studies were performed to clinically validate the REMS technique. In 2019, a multicenter study performed in Italy by Di Paola et al. evaluated the REMS precision and accuracy on a large population of 1914 postmenopausal women enrolled in six clinical centers [30]. REMS accuracy was assessed by comparing US-BMD results with BMD by DXA, the reference technique in clinical practice. Regarding the intraoperator precision (two consecutive acquisitions, same patient, same operator), a root-mean-square coefficient of variation (RMS-CV) of 0.38% was found at lumbar spine, while 0.32% was found at femoral neck. Regarding the inter-operator repeatability (a third REMS scan on the same patient, performed by a second operator), RMS-CV values of 0.54% and 0.48% were reported for lumbar spine and femoral neck, respectively [30]. A very recent study from Messina et al. in 2023 confirmed these optimal values of intraoperator precision (RMS-CV: 0.47% for lumbar spine, 0.32% for femoral neck), as well as those related to the inter-operator repeatability (0.55% for lumbar spine, 0.51% for femoral neck) [31]. Such values are generally superior to those of DXA, as the precision of this technique has been reported to range between 0.91 to 1.92% at the spine and 1.5 to 2.25% at the femoral neck [32, 33].

Diagnostic accuracy of REMS was mainly assessed by comparing it to DXA and determining the concordance between these two techniques. The Italian multicenter study by Di Paola et al. estimated the concordance between REMS and DXA by calculating the Cohen’s kappa (k), reporting a good discrimination capability between osteopenic, normal, and osteoporotic subjects with *k* = 0.82 (lumbar spine) and *k* = 0.79 (femoral neck) [30]. Additional analysis showed that the sensitivity of REMS technique in differentiating osteoporotic from non-osteoporotic (osteopenic and healthy) subjects was 91.7% at the spine and 91.5% at the femur, with a corresponding specificity of 92.0% and 91.8%, respectively. Authors also evaluated the degree of correlation between the two techniques, reporting very high values with *r* = 0.94 for the spine and *r* = 0.93 for the femoral neck, both values being statistically significant [30].

In 2021, a wider European multicenter study was conducted in five clinical centers to evaluate the diagnostic accuracy of REMS technique compared to DXA for femoral neck and lumbar spine [34]. The study involved 4307 female Caucasian subjects, and the comparison in terms of diagnostic classification of patients with/without osteoporosis showed a sensitivity of 90.4% and a specificity of 95.5% at femoral neck, while the diagnostic accuracy yielded a sensitivity of 90.9% with a specificity of 95.1% at lumbar spine [34]. Such results indicated a very good sensitivity and specificity for REMS in identifying osteoporotic patient in terms of agreement with DXA.

An interesting aspect is the possibility of REMS technology to overcome those artifacts commonly encountered (osteoarthrosis, vertebral fractures) that typically overestimate BMD values at DXA. Caffarelli et al. showed that in subjects with spinal osteoarthritis, REMS was able to identify a greater number of subjects as “osteoporotic” compared with DXA (35.1% vs 9.3%, respectively) [35]. Further studies will better clarify this ability, possibly by comparing REMS values with those of other techniques such as quantitative CT.

The use or REMS has been explored with promising results in several conditions leading to secondary osteoporosis, such as in young women with anorexia nervosa [36] or in elderly subjects with type 2 diabetes mellitus [37], as well as conditions in which DXA assessment is not feasible such as in healthy pregnant women [38].

A recent development of REMS is the Fragility Score. This score (adimensional value between 0 and 100) is obtained by comparing the patient’s spectrum with reference models of fractured and not fractured subjects and is an indicator of bone quality independent of BMD: Lower values are associated with a good quality of examined bone and vice versa. Data about FS are growing, with the results obtained from an up to 5-year longitudinal study by Pisani et al. showing how lumbar spine and femoral FS are able to discriminate between fractured and non-fractured subjects with higher performance with respect to T-scores values measured by both DXA and REMS [29]. Promising results for FS have also been reported in differentiating between subjects with primary and disuse-related osteoporosis [39].

Regarding some limitations related to REMS, of course there may be variability in its performance across different patient populations or clinical settings [40]. Of course, other limitations may arise from those conditions that usually limit the usefulness of ultrasound (obesity, operator's skills, or other conditions that may create an obstacle to ultrasound beam such as air); nevertheless, the technique is capable to get proper measurements even from small portions of the examined vertebra; therefore, a diagnostic value is obtained even in the presence of obstacle to full vertebra visualization. In any case, fasting is suggested before the exam as for any other abdominal ultrasound scan. Regarding operator’s skills, the very good inter-operator reproducibility suggests that, after proper machine training, REMS it is little affected.

Lastly, the accuracy of REMS was compared only with DXA, which represents the gold standard. Further studies are comparing REMS accuracy with that of more complex techniques such as QCT.

## Body composition analysis using DXA

The study of body composition has been receiving increasing attention from the scientific community in recent years, becoming a trending topic in medicine. Body composition analysis refers to the description and quantification of the different components that constitute the human body [41]. Body composition analysis can be applied to the assessment of physiological and paraphysiological conditions like aging or adaptation to training in athletes, but also to the evaluation of a myriad of diseases such as obesity, diabetes mellitus, cancer, and sarcopenia [42–44].

Several imaging and non-imaging techniques are indeed available for body composition analysis, with variable characteristics (see Table 1). Non-imaging techniques include anthropometry and bioimpedance analysis (BIA). Imaging methods include DXA, CT, MRI, and US [45]. At present, DXA is the most frequent radiologic technique used to evaluate body composition, being accurate, widely available, and associated with a very low radiation dose [46].

**Table 1:** Summary of features characterizing the imaging techniques used for body composition analysis; adapted from [45, 46]

Feature	DXA	CT	MRI	US
Radiation	+	+ + +	−	−
Cost	+	+ +	+ + +	+
Availability	+ + +	+ +	+	+ + +
Precision	+ + +	+ + +	+ + +	+
Portability	−	−	−	+ +
Complexity	+	+ + +	+ + +	+ +
Three-dimensional assessment	−	+ + +	+ +	−
Dynamic evaluation	−	−	−	+ + +
Distinction of visceral versus subcutaneous fat	+	+ + +	+ + +	+
Distinction of intramuscular fat	−	+ +	+ + +	+
Opportunistic use	−	+ + +	+	−

*DXA* dual-energy X-ray absorptiometry, *CT* computed tomography, *MRI *magnetic resonance imaging, *US *ultrasound imaging

DXA employs two X-ray beams of different energy. The attenuation of X-ray beams through the body depends on the density and thickness of human tissues but also on the energy of photons. DXA thus measures the *R*-value, which is the ratio of the attenuation coefficients at the two different photon energy levels. The *R*-value is specific to each tissue. It is constant for bone and fat in all individuals, while showing inter-individual variability for soft tissue, depending on its composition. A higher fat percentage corresponds to a lower *R*-value [47]. DXA is based on a three-compartmental model, comprising fat mass (FM), non-bone lean mass (LM), and bone mineral content (BMC) [47]. Of note, DXA does not measure directly these three components. In pixels that contain bone, this technique distinguishes bone from soft tissue, including both FM and LM. In order to quantify separately LM and FM, DXA relies on pixels adjacent to bone that comprise only soft tissue [48]. In clinical practice, whole-body DXA is generally performed for the study of body composition. The effective dose for a whole-body scan is only 4–5 μSv with the newest generation of densitometers, meaning that DXA can be considered a low-dose technique for both patients and operators [49, 50].

Body composition parameters obtained by DXA are susceptible to error or variation due to technical and/or biological aspects [49]. To minimize biological variability, it is recommended to acquire the scan in standardized conditions, preferably with subjects at rest and euhydrated, after an overnight fast, with an empty bladder [51].

**Fig. 5:**
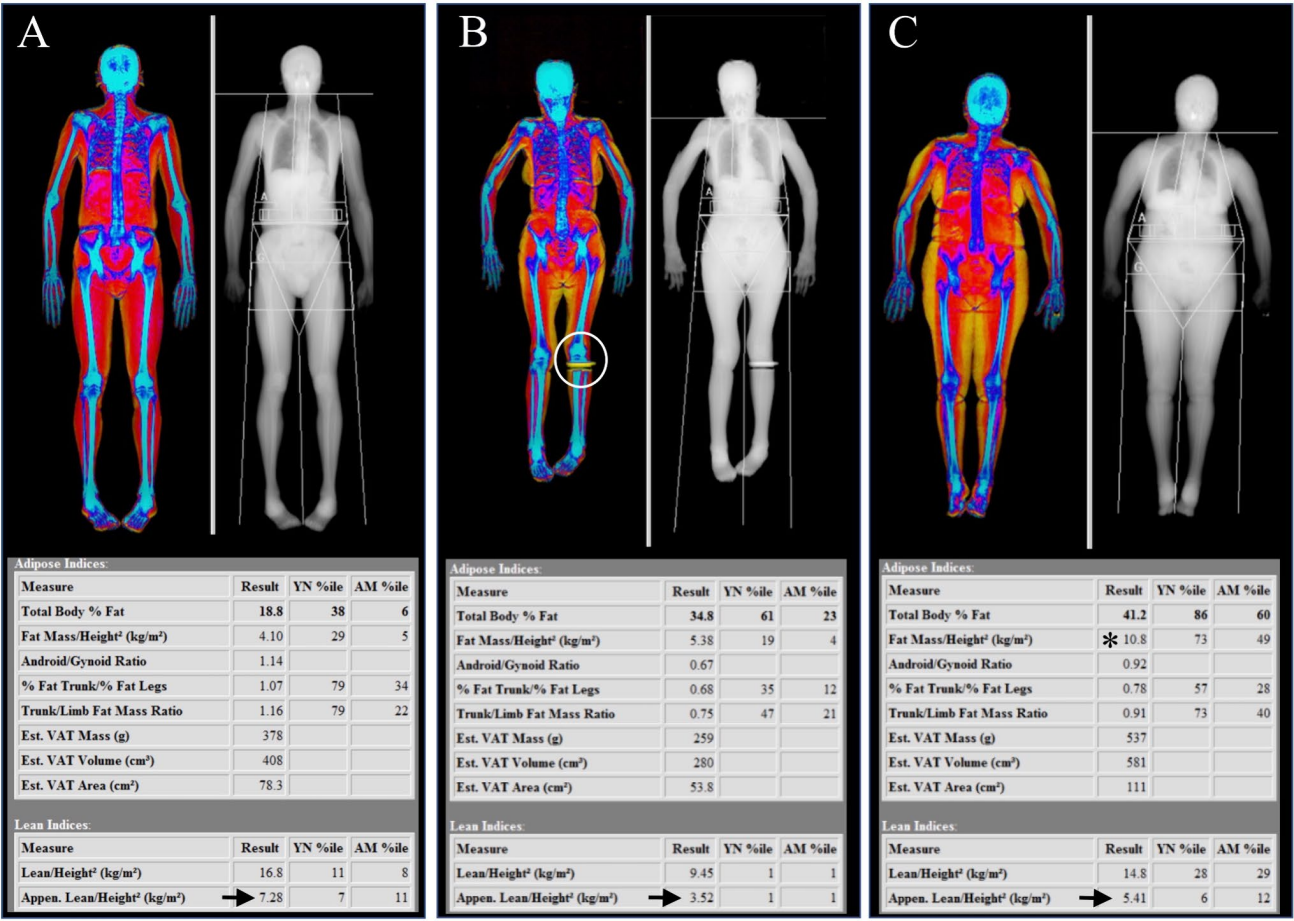
The use of total body DXA for evaluating different conditions using lean and adipose indices of body composition. Figure A shows a 64-year-old man with normal values of appendicular lean mass index (ALMI), corresponding to 7.28 kg/m^2^ (normal values for men ≥ 7.0 kg/m^2^ [54]). Figure B shows a 80-year-old woman with severe sarcopenia as demonstrated by the very low values of ALMI (3.52 kg/m^2^, arrow); normal values for women are superior or equal to 6.0 kg/m^2^ [54]. The presence of metal implants at the level of left tibia can also be appreciated (see circle). Figure C shows the case of a 74-year-old woman diagnosed with sarcopenia and excessive body fat, predisposing to the condition called “sarcopenic obesity.” ALMI value is below 6.0 kg/m^2^ (see arrow), with concurrent fat mass index (FMI) of 10.8 kg/m^2^. FMI ≥ 9.0 kg/m^2^ for women is considered as excessive fat [79]

The report of a whole-body DXA scan for body composition analysis should include values of body mass index (BMI), bone mineral density (BMD), bone mineral content (BMC), total mass, total lean mass, total fat mass, and percent fat mass [45]. DXA also allows to quantify visceral adipose tissue (VAT) by the use of specific algorithms that are integrated in the newest software versions (CoreScan™, GE Healthcare or InnerCore™, Hologic) [49]. These algorithms employ geometric assumptions to subtract the mass of the subcutaneous adipose tissue (SAT) layer from the total fat mass in the android region, as the width of the SAT layer can be estimated along the lateral borders of the abdomen [52]. In addition, DXA enables to obtain several body composition indices, such as android/gynoid (AG) ratio, fat mass index (FMI = fat mass/height squared), lean mass index (LMI = lean mass/height squared), appendicular lean mass index (ALMI = appendicular lean mass/height squared; also called appendicular skeletal muscle index or ASMI), and lipodystrophy indices (trunk to leg fat mass ratio and trunk to leg percent fat) [45]. These indices can be easily computed by inserting anthropometric parameters like weight and height in the system, and by defining a number of standard regions of interest (ROI) during the analysis of the DXA scan, including the trunk, arm, leg, android, and gynoid areas [47]. Their main problem is that the respective cutoff values are often variably defined in literature, with no universal consensus. However, adiposity indices can improve the stratification of cardiometabolic risk beyond BMI [53]. In addition, since BMI does not differentiate between lean mass and fat mass, the evaluation of obesity may benefit from the use of adiposity indices derived by DXA, including percent fat mass and FMI. By contrast, lean mass indices are now being employed for the assessment and diagnosis of sarcopenia [46]. The revised European consensus on definition and diagnosis of sarcopenia by the European Working Group on Sarcopenia in Older People (EWGSOP) supports the use of DXA to determine low muscle quantity and recommends specific cutoff points for appendicular skeletal mass (ASM) and ASMI; these are appendicular skeletal mass < 20 kg in men and < 15 kg in women and ASMI < 7 kg/m^2^ in men and < 5.5 kg/m^2^ in women [54]. Of note, these recommendations focus on the European population, but there may be relevant differences in body composition parameters depending on ethnicity, although this holds true more for adiposity indices [55]. Figure 5 shows three cases of total body DXA evaluating different conditions using body composition parameters (lean and adipose indices).

DXA has been compared with CT and MRI for the measurement of fat mass and skeletal mass. CT and MRI are generally considered the gold standard in body composition analysis, particularly at organ/tissue level [56]. While DXA may underestimate fat mass and overestimate lean mass as compared, respectively, to CT/MRI-measured adipose tissue and skeletal muscle mass, since it considers chemical compartments (= FM as lipids and LM as water, proteins, carbohydrates, and other soft tissue components including minerals) rather than actual anatomical compartments [46], DXA-derived parameters seem to correlate well with CT- and MRI-derived measurements [57].

It is worth underlining that DXA can provide simultaneous information on soft tissues and bone mineral density/content. This is relevant in the diagnosis of an emerging geriatric syndrome associated with adverse outcomes known as osteosarcopenia, which typically requires the presence of both osteopenia/osteoporosis and sarcopenia [58]. Of note, osteosarcopenia does not have a dedicated diagnostic model, which rather stems from those of its two respective components. Therefore, its definition is not completely standardized: While some published studies include only osteoporosis as part of osteosarcopenia, others also include osteopenia; in addition, several diagnostic criteria can be used for sarcopenia [59]. Osteopenia/osteoporosis are diagnosed based on WHO criteria which rely on the T-score derived from DXA-measured aBMD [60]. According to the EWGSPO2 algorithm [54], which is widely accepted in Europe, confirmed sarcopenia instead requires the presence of low muscle strength together with low muscle quality or quantity; the latter can be identified via low ASM or ASMI determined through DXA. DXA is unique in its ability of addressing both components of the osteosarcopenic syndrome.

To sum up, despite having some limitations, DXA appears to be an excellent option for the evaluation of body composition in clinical practice, given its low dose, wide availability, limited cost, and good precision and accuracy. A whole-body DXA scan provides a considerable amount of information with major clinical relevance. However, further research is needed to optimize cutoff points for fat mass and lean mass indices.

## Abdominal aortic calcification assessment using DXA

Abdominal aortic calcification (AAC) is a common pathologic condition leading to the formation of calcific deposits in the wall of the abdominal aorta. The prevalence of AAC increases with advancing age, reaching 100% in men and women over age 75 [61]. AAC is associated with conventional cardiovascular risk factors such as hypertension, smoking, dyslipidemia, hyperglycemia, and overweight/obesity [62]. On the other hand, AAC is now recognized as an independent predictor of cardiovascular disease and mortality [63], in terms of coronary heart disease and myocardial infarction [64] as well as stroke and intermittent claudication [65]. Therefore, detection of AAC, together with assessment of its extent and severity, may improve the stratification of cardiovascular risk in the general population, which could eventually translate into optimized clinical management and prevention of cardiovascular events [66]. In addition, an independent association of AAC with decreased bone mineral density (BMD) and increased fracture risk has been reported by multiple studies [67, 68] and confirmed by a recent meta-analysis [69]. This suggests that the evaluation of AAC may simultaneously provide information on the risk of cardiovascular morbidity/mortality and bone loss.

AAC is detectable using different radiologic techniques, including lateral lumbar radiographs, lateral spine DXA, and CT [70]. DXA is commonly performed in middle-aged and elderly individuals for the diagnosis and follow-up of osteoporosis, typically via posteroanterior spine and hip scans. Although this is not routinely performed in current clinical practice, AAC can be correctly identified and scored on an additional lateral spine DXA image intended for vertebral fracture assessment (VFA), thanks to the improved spatial resolution of modern DXA devices [71, 72].

The severity of AAC can be graded using two validated scoring systems. The first scoring system—a 24-point semiquantitative method (also known as AAC-24)—considers calcifications in the abdominal aorta at the levels corresponding to L1–L4 vertebrae [73]. A score from 0 to 3 is attributed to calcific lesions in the anterior and posterior aortic walls in the eight segments, giving a maximum of 24 points, as shown in Table 2. A simplified 8-point semiquantitative method (also known as AAC-8) was proposed in 2006 [74]. This scoring system considers the aggregate length of calcification separately for the anterior and posterior aortic walls corresponding to L1–L4, as if the calcific deposits at different levels were stacked end to end. A score from 0 to 4 is attributed to the anterior and posterior aortic walls as shown in Table 2, for a maximum of 8 points. Both scoring systems can be applied to lateral lumbar radiographs as well as lateral spine DXA images obtained for VFA. A score ≥ 5 points for AAC-24 and ≥ 3 points for AAC-8 identifies patients at risk for cardiovascular disease [75, 76].

**Table 2:** Two scoring systems (Kauppila and Schousboe) regarding the anterior and posterior calcification of aortic walls at L1-L4 segments

AAC-24 scoring system for grading the severity of calcific lesions in the anterior and posterior aortic walls of each vertebral segment (L1–L4); adapted from Kauppila et al. [73]	
Score	Description
0	No calcification
1	Calcification filling < 1/3 of the longitudinal wall of the aorta
2	Calcification filling ≥ 1/3 but < 2/3 of the longitudinal wall of the aorta
3	Calcification filling ≥ 2/3 of the longitudinal wall of the aorta

AAC-8 scoring system for grading the severity of calcifications in the anterior and posterior aortic walls at L1–L4; adapted from Schousboe et al. [74]	
Score	Description
0	No calcification
1	Aggregate length of calcification ≤ 1 vertebral height
2	Aggregate length of calcification > 1 but ≤ 2 vertebral heights
3	Aggregate length of calcification > 2 but ≤ 3 vertebral heights
4	Aggregate length of calcification > 3 vertebral heights

**Fig. 6:**
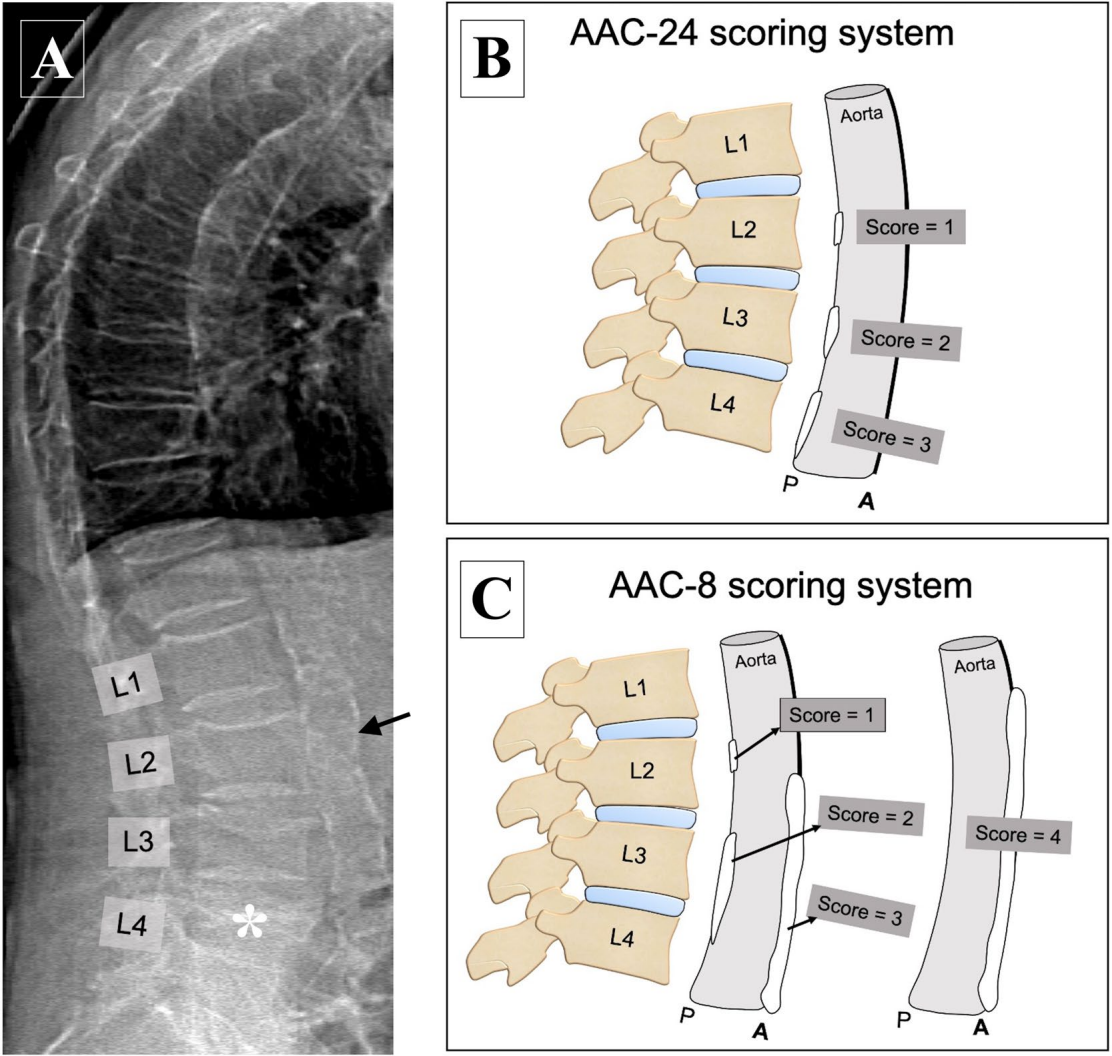
Image A shows an example of a lateral DXA spine image obtained for vertebral fracture assessment (VFA) that can be used for scoring abdominal aortic calcification (AAC). Extensive calcification can be appreciated both in the anterior and posterior aortic wall at L1–L4 levels (arrow), with a maximum score both for AAC-24 and AAC-8. Multiple fragility fractures of various degree can be seen at dorsal and lumbar spine, with a severe crush fracture at L4 (asterisk). Image B and C show a schematic representation of AAC-24 and AAC-8 scoring systems, respectively, to diagnose aortic wall calcifications in the anterior and posterior walls. The AAC-24 system is based on a score from 0 to 3 at each level, reaching a maximum of 24 points. The AAC-8 system is based on a score from 0 to 4 overall, for a total of 8 points considering both anterior and posterior wall

The performance of DXA in the detection and scoring of AAC has been evaluated in comparison with lateral lumbar radiographs and CT, which may be set as gold standards [77]. Findings from selected studies are summarized in Table 3. Overall, available evidence indicates a good correlation with radiographs and a moderate correlation with CT. An example of AAC estimation with the use of lateral DXA is shown in Fig. 6, together with a schematic representation of AAC-24 and AAC-8 scoring systems.

**Table 3:** Studies comparing the diagnostic performance of DXA versus lumbar radiographs or CT in the detection and scoring of AAC

Study author (year) [reference]	Study population	Study design	Results
Schousboe et al. (2006) [74]	57 elderly women (AAC substudy) Age ≥ 65 years (mean 76.1 [95% CI: 74.1–78.1] Mean BMI 25.7 kg/m^2^ [95% CI: 24.4–27.0]	Lateral DXA images and lateral lumbar radiographs performed and assessed by two readers Scoring with AAC-24 and AAC-8	Mean AAC-24: - Radiographs: 5.89 (reader 1) and 6.37 (reader 2) - DXA: 4.73 (reader 1) and 5.25 (reader 2) Intraclass correlation coefficient (ICC): - DXA versus radiographs: - 0.81 [95% CI: 0.66–0.90] (reader 1) - 0.82 [95% CI: 0.69–0.90] (reader 2) - Reader 1 versus 2 for radiographs: 0.92 [95% CI: 0.88–0.95] - Reader 1 versus 2 for DXA: 0.89 [95% CI: 0.80–0.94] Ability of DXA to distinguish AAC-24 score ≥ 5 on standard radiographs set as gold standard: - ROC curve area: - 0.83 [95% CI 0.71–0.97] (reader 1) - 0.88 [95% CI 0.79–0.97] (reader 2) - Sensitivity: 65% (reader 1) and 78% (reader 2) - Specificity: 90% (reader 1) and 87% (reader 2) AAC-8 versus AAC-24 (reader 1 only): - Correlation coefficient: - 0.93 [95% CI 0.88–0.96] for DXA - 0.95 [95% CI 0.89–0.97] for radiographs - ROC curve area for prediction of AAC-24 > 5: - 0.98 [95% CI: 0.95–1.0] for DXA - 0.95 [95% CI: 0.91–0.99] for radiographs - ROC curve area for ability of AAC-8 to distinguish AAC-24 score ≥ 5 versus < 5 versus standard radiographs set as gold standard: 0.78 [95% CI: 0.66–0.91] * Acceptable predictive accuracy in ROC curve areas: 0.70–0.90 * Superb predictive accuracy in ROC curve areas: > 0.90
Schousboe et al. (2007) [75]	156 postmenopausal women Age ≥ 55 years (mean: 68.7) Mean BMI: 27.0 kg/m^2^ 94.8% White and 5.2% Black ethnicity Prevalent vertebral fracture index (PVFI) ≥ 3 or = 2 with femoral neck T-score ≤—2.5 SD	Lateral radiographs and VFA images performed and evaluated by one reader (VFA images re-read using AAC-8 after a few months) Scoring with AAC-24 and AAC-8	Intraclass correlation coefficient (ICC): - VFA versus radiographs for AAC-24: 0.80 [95% CI: 0.68–0.87] - VFA versus radiographs for AAC-8: 0.76 [95% CI: 0.65–0.84] Ability to distinguish AAC-24 score ≥ 5 on standard radiographs set as gold standard: - ROC curve area: 0.95 [95% CI: 0.91–0.99] for radiographs using AAC-8 - ROC curve area: 0.85 [95% CI: 0.77–0.94] for VFA using AAC-24 - ROC curve area: 0.84 [95% CI: 0.76–0.92] for VFA using AAC-8 Diagnostic performance in identification of AAC-24 score ≥ 5 on standard radiographs set as gold standard: - AAC-24 ≥ 5 for VFA: sensitivity 59% and specificity 97% - AAC-8 ≥ 3 for VFA: sensitivity 62% and specificity 96% Agreement with AAC-24 ≥ 5 on standard radiographs: - Moderate for VFA using AAC-24 ≥ 5 (kappa = 0.62 [95% CI: 0.55–0.70]) - Moderate for VFA using AAC-8 ≥ 3 (kappa = 0.63 [95% CI: 0.55–0.71]) Agreement between initial and repeat AAC-8 scores for VFA: - Good (kappa = 0.87 [95% CI: 0.80–0.92]) * Acceptable predictive accuracy in ROC curve areas: 0.70–0.90 * Superb predictive accuracy in ROC curve areas: > 0.90
Bazzocchi et al. (2012) [77]	75 patients (30 women, 40 men) Age 59.3 ± 9.7 years BMI 24.8 ± 5.8 kg/m^2^	Digital lateral lumbar radiographs (DR) and VFA images performed and evaluated independently by 2 radiologists (1 with 15 years of experience and 1 with 5 years of experience); evaluation repeated after 7 days for the most experienced radiologist Scoring with AAC-24 and AAC-8	AAC detection (VFA versus DR): - Sensitivity 78.6% - Specificity 85.7% - Accuracy 81.0% CVD risk assessment with AAC-24 (VFA versus DR): - Sensitivity 86.7% - Specificity 100% - Accuracy 96.8% CVD risk assessment with AAC-8 (VFA versus DR): - Sensitivity 86.7% - Specificity 93.8% - Accuracy 92.1% Intraobserver agreement: - Excellent for AAC detection (kappa = 1.00 for both DR and VFA) and CVD risk assessment (kappa = 0.96 and 1.00 for VFA versus 0.95 and 0.96 for VFA using AAC-24 and AAC-8, respectively) - No significant difference between DR and VFA Inter-observer agreement: - Good for AAC detection (kappa = 0.76 for DR versus 0.71 for VFA) - Excellent for CVD risk assessment (kappa = 0.91 and 0.87 for DR versus 0.85 and 0.83 for VFA using AAC-24 and AAC-8, respectively) - No significant difference between DR and VFA
Setiawati et al. (2016) [70]	323 postmenopausal women Age 23–93 years (mean: 64.68 ± 0.65 SD)	Digital lateral lumbar radiographs (DR), VFA images, and QCT performed and evaluated by an experienced radiologist (25 years of experience) Scoring with AAC-8	Mean AAC-8 scores: - DR: 1.79 ± 0.13 SD - DXA: 1.41 ± 0.11 SD - QCT: 1.90 ± 0.12 SD Intraclass correlation coefficient (ICC): - DXA versus DR: 0.699 [95% CI: 0.638–0.752] - QCT versus DR: 0.829 [95% CI: 0.783–0.865] ROC curve area versus DR set as gold standard: - DXA: 0.826 [predictive accuracy: 0.764–0.888] - QCT: 0.948 [predictive accuracy: 0.922–0.974]
Cecelja et al. (2013) [78]	105 healthy women Age 64.2 ± 7.4 years	Lateral DXA images and non-contrast abdominal CT performed Scoring with AAC-24 and AAC-8 for DXA Analysis with total calcium volume score for CT (aortic calcification defined as any area within the aorta > 1 mm^2^ with attenuation ≥ 130 HU)	Mean volume score for CT: - 0.20 ± 0.41 cm^2^ Mean AAC-24 score for DXA: - 2.39 ± 3.91 Mean AAC-8 score for DXA: - 1.47 ± 2.13 Correlation between AAC-24 and AAC-8: - Very high (r = 0.98) Agreement between DXA and CT: - Moderate (r = 0.58) for both AAC-24 and AAC-8 Diagnostic performance of DXA versus CT set as gold standard: - AAC > 0 cm^3^ on CT (first tertile): - Sensitivity 56% - Specificity 80% - AUC 0.68 [95% CI: 0.55–0.80] - AAC > 000.8 cm^3^ on CT (second tertile): - Sensitivity 64% - Specificity 84% - AUC 0.74 [95% CI: 0.65–0.84] - AAC > 00.8 cm^3^ on CT (third tertile): - Sensitivity 71% - Specificity 63% - AUC 0.67 [95% CI: 0.56–0.78]

*AAC-24* 24-point semiquantitative scoring system for AAC, *AAC-8* 8-point semiquantitative scoring system for AAC, *AAC* abdominal aortic calcification, *BMI* body mass index, *CI* confidence interval, *CT* computed tomography, *DR* digital radiographs, *DXA* dual-energy X-ray absorptiometry, *HU* Hounsfield units, *ROC* receiver operating characteristic, *VFA* vertebral fracture assessment

To conclude, lateral DXA images obtained for VFA allow detection and grading of AAC with acceptable sensitivity and specificity compared to lateral radiographs or CT, as well as good repeatability and reproducibility. Although lateral radiographs and CT/QCT show superior accuracy, DXA is a valuable option to assess AAC in clinical practice, in light of its low dose (≤ 0.005 mSv for a VFA scan [50]) and wide availability. Lateral DXA may be intended as a sort of screening tool for AAC detection in individuals who do not have manifestations of CVD [70]. Patients at risk of CVD according to the severity of AAC on lateral spine DXA images may benefit from a tighter control of cardiovascular risk factors, although no specific therapeutic interventions are currently available for AAC [64].

## Conclusion

Osteoporosis and the consequent risk of fracture still have a high impact on healthcare systems and patients’ quality of life. New tools and diagnostic technique have been developed in recent years to further refine the fracture risk estimation.

DXA can be considered as a “multiparametric” technique, still representing the reference standard for assessing conditions such as sarcopenia and osteoporosis. The use of lateral DXA may allow not only the identification of vertebral fractures, but also allow the evaluation of aortic calcifications. Additional DXA-based software can also explore parameters that goes beyond BMD, such as the Trabecular Bone Score and the Bone Strain Index, providing indirect data about bone microarchitecture and bone strength, respectively.

At the same time, REMS technology emerged in the last years as a novel and accurate ultrasound-based tool to estimate BMD. Scientific evidence confirms that REMS has very good concordance with DXA, being a predictor of fracture risk in postmenopausal women. Literature about REMS is growing also in the context of other secondary osteoporosis conditions. Finally, the use of REMS Fragility Score offers a further parameter to assess skeletal fragility independently from BMD.

## References

[1] Kanis JA, Cooper C, Rizzoli R, Reginster JY (2019) European guidance for the diagnosis and management of osteoporosis in postmenopausal women. Osteoporos Int 30:3–44. 10.1007/s00198-018-4704-5 30324412 10.1007/s00198-018-4704-5 PMC7026233

[2] Miller PD, Siris ES, Barrett-Connor E et al (2002) Prediction of fracture risk in postmenopausal white women with peripheral bone densitometry: evidence from the national osteoporosis risk assessment. J Bone Miner Res 17:2222–2230. 10.1359/jbmr.2002.17.12.2222 12469916 10.1359/jbmr.2002.17.12.2222

[3] Adams JE (2013) Advances in bone imaging for osteoporosis. Nat Rev Endocrinol 9:28–42. 10.1038/nrendo.2012.217 23232496 10.1038/nrendo.2012.217

[4] Hans D, Barthe N, Boutroy S et al (2011) Correlations between trabecular bone score, measured using anteroposterior dual-energy X-ray absorptiometry acquisition, and 3-dimensional parameters of bone microarchitecture: an experimental study on human cadaver vertebrae. J Clin Densitom 14:302–312. 10.1016/j.jocd.2011.05.005 21724435 10.1016/j.jocd.2011.05.005

[5] Silva BC, Broy SB, Boutroy S et al (2015) Fracture risk prediction by non-BMD DXA measures: the 2015 ISCD official positions part 2: trabecular bone score. J Clin Densitom 18:309–330. 10.1016/j.jocd.2015.06.008 26277849 10.1016/j.jocd.2015.06.008

[6] Bousson V, Bergot C, Sutter B et al (2012) Trabecular bone score (TBS): available knowledge, clinical relevance, and future prospects. Osteoporos Int 23:1489–1501. 10.1007/s00198-011-1824-6 22083541 10.1007/s00198-011-1824-6

[7] McCloskey EV, Odén A, Harvey NC et al (2016) A meta-analysis of trabecular bone score in fracture risk prediction and its relationship to FRAX. J Bone Miner Res 31:940–948. 10.1002/jbmr.2734 26498132 10.1002/jbmr.2734

[8] Krohn K, Schwartz EN, Chung YS, Lewiecki EM (2019) Dual-energy X-ray absorptiometry monitoring with trabecular bone score: 2019 ISCD official position. J Clin Densitom 22:501–505 31383412 10.1016/j.jocd.2019.07.006

[9] Bandirali M, Poloni A, Sconfienza LM et al (2015) Short-term precision assessment of trabecular bone score and bone mineral density using dual-energy X-ray absorptiometry with different scan modes: an in vivo study. Eur Radiol. 10.1007/s00330-015-3606-6 25663312 10.1007/s00330-015-3606-6

[10] Shevroja E, Aubry-Rozier B, Hans G et al (2019) Clinical performance of the updated trabecular bone score (TBS) algorithm, which accounts for the soft tissue thickness: the Osteolaus study. J Bone Miner Res 34:2229–2237. 10.1002/jbmr.3851 31419331 10.1002/jbmr.3851

[11] Haeri NS, Perera S, Ferreiro I et al (2022) Trabecular bone score in the hip: a new method to examine hip bone microarchitecture-a feasibility study. Arch Osteoporos 17:126. 10.1007/s11657-022-01168-9 36125566 10.1007/s11657-022-01168-9 PMC12276939

[12] Ulivieri FM, Rinaudo L (2021) Beyond bone mineral density: a new dual X-ray absorptiometry index of bone strength to predict fragility fractures, the bone strain Index. Front Med (Lausanne) 7 10.3389/fmed.2020.590139 PMC7843921 33521014

[13] Ulivieri FM, Rinaudo L (2022) The bone strain index: an innovative dual X-ray absorptiometry bone strength index and its helpfulness in clinical medicine. J Clin Med 11 10.3390/jcm11092284 PMC9102586 35566410

[14] Bazzocchi A, Isaac A, Dalili D et al (2022) Imaging of metabolic bone diseases: the spine view, part I. Semin Musculoskelet Radiol 26:478–490. 10.1055/s-0042-1754340 36103889 10.1055/s-0042-1754340

[15] Colombo C, Libonati F, Rinaudo L et al (2019) A new finite element based parameter to predict bone fracture. PLoS ONE. 10.1371/journal.pone.0225905 31805121 10.1371/journal.pone.0225905 PMC6894848

[16] Han KS, Rohlmann A, Zander T, Taylor WR (2013) Lumbar spinal loads vary with body height and weight. Med Eng Phys 35:969–977. 10.1016/j.medengphy.2012.09.009 23040051 10.1016/j.medengphy.2012.09.009

[17] Terzini M, Aldieri A, Rinaudo L et al (2019) Improving the hip fracture risk prediction through 2D finite element models from DXA images: validation against 3D models. Front Bioeng Biotechnol 7:220. 10.3389/fbioe.2019.00220 31552243 10.3389/fbioe.2019.00220 PMC6746936

[18] Ulivieri FM, Rinaudo L, Messina C et al (2022) Bone Strain Index: preliminary distributional characteristics in a population of women with normal bone mass, osteopenia and osteoporosis. Radiol Med 127:1151–1158. 10.1007/s11547-022-01543-z 36057931 10.1007/s11547-022-01543-z

[19] Sornay-Rendu E, Duboeuf F, Ulivieri FM et al (2022) The bone strain index predicts fragility fractures. The OFELY study. Bone. 10.1016/j.bone.2022.116348 35121211 10.1016/j.bone.2022.116348

[20] Ulivieri FM, Piodi LP, Rinaudo L et al (2020) Bone strain index in the prediction of vertebral fragility refracture. Eur Radiol Exp 4:23. 10.1186/s41747-020-00151-8 32274595 10.1186/s41747-020-00151-8 PMC7145882

[21] Messina C, Rinaudo L, Cesana BM et al (2021) Prediction of osteoporotic fragility re-fracture with lumbar spine DXA-based derived bone strain index: a multicenter validation study. Osteoporos Int 32:85–91. 10.1007/s00198-020-05620-9 32936366 10.1007/s00198-020-05620-9

[22] Tabacco G, Naciu AM, Messina C et al (2021) DXA-based bone strain index: a new tool to evaluate bone quality in primary hyperparathyroidism. J Clin Endocrinol Metab 106:2304–2312. 10.1210/clinem/dgab317 33963754 10.1210/clinem/dgab317 PMC8599893

[23] Tabacco G, Naciu AM, Messina C et al (2023) DXA-based bone strain index in normocalcemic primary hyperparathyroidism. Osteoporos Int 34:999–1003. 10.1007/s00198-023-06669-y 36640186 10.1007/s00198-023-06669-y

[24] Rodari G, Scuvera G, Ulivieri FM et al (2018) Progressive bone impairment with age and pubertal development in neurofibromatosis type I. Arch Osteoporos 13:93. 10.1007/s11657-018-0507-8 30151698 10.1007/s11657-018-0507-8

[25] Messina C, Piodi LP, Grossi E et al (2020) Artificial neural network analysis of bone quality DXA parameters response to teriparatide in fractured osteoporotic patients. PLoS ONE 15:e0229820. 10.1371/journal.pone.0229820 32160208 10.1371/journal.pone.0229820 PMC7065795

[26] Casciaro S, Peccarisi M, Pisani P et al (2016) An advanced quantitative echosound methodology for femoral neck densitometry. Ultrasound Med Biol 42:1337–1356. 10.1016/j.ultrasmedbio.2016.01.024 27033331 10.1016/j.ultrasmedbio.2016.01.024

[27] Conversano F, Franchini R, Greco A et al (2015) A novel ultrasound methodology for estimating spine mineral density. Ultrasound Med Biol 41:281–300. 10.1016/j.ultrasmedbio.2014.08.017 25438845 10.1016/j.ultrasmedbio.2014.08.017

[28] Diez-Perez A, Brandi ML, Al-Daghri N et al (2019) Radiofrequency echographic multi-spectrometry for the in-vivo assessment of bone strength: state of the art—outcomes of an expert consensus meeting organized by the European Society for Clinical and Economic Aspects of Osteoporosis, Osteoarthritis and Mus. Aging Clin Exp Res 31:1375–1389 31422565 10.1007/s40520-019-01294-4 PMC6763416

[29] Pisani P, Greco A, Conversano F et al (2017) A quantitative ultrasound approach to estimate bone fragility: a first comparison with dual X-ray absorptiometry. Measurement (Lond) 101:243–249. 10.1016/j.measurement.2016.07.033 10.1016/j.measurement.2016.07.033

[30] Di Paola M, Gatti D, Viapiana O et al (2019) Radiofrequency echographic multispectrometry compared with dual X-ray absorptiometry for osteoporosis diagnosis on lumbar spine and femoral neck. Osteoporos Int 30:391–402. 10.1007/s00198-018-4686-3 30178159 10.1007/s00198-018-4686-3

[31] Messina C, Gitto S, Colombo R et al (2023) Short-term precision and repeatability of radiofrequency echographic multi spectrometry (REMS) on lumbar spine and proximal femur: an in vivo study. J Imaging. 10.3390/JIMAGING9060118 37367466 10.3390/JIMAGING9060118 PMC10300972

[32] Messina C, Buonomenna C, Menon G et al (2019) Fat mass does not increase the precision error of trabecular bone score measurements. J Clin Densitom 22:359–366. 10.1016/j.jocd.2019.01.001 30661747 10.1016/j.jocd.2019.01.001

[33] Messina C, Acquasanta M, Rinaudo L et al (2021) Short-term precision error of bone strain index, a new DXA-based finite element analysis software for assessing hip strength. J Clin Densitom 24:330–337. 10.1016/j.jocd.2020.10.013 33199190 10.1016/j.jocd.2020.10.013

[34] Cortet B, Dennison E, Diez-Perez A et al (2021) Radiofrequency echographic multi spectrometry (REMS) for the diagnosis of osteoporosis in a European multicenter clinical context. Bone 143:115786. 10.1016/j.bone.2020.115786 33278653 10.1016/j.bone.2020.115786

[35] Caffarelli C, Tomai Pitinca MD, Al Refaie A et al (2022) Could radiofrequency echographic multispectrometry (REMS) overcome the overestimation in BMD by dual-energy X-ray absorptiometry (DXA) at the lumbar spine? BMC Musculoskelet Disord. 10.1186/s12891-022-05430-6 35590362 10.1186/s12891-022-05430-6 PMC9118880

[36] Caffarelli C, Al Refaie A, De Vita M et al (2022) Radiofrequency echographic multispectrometry (REMS): an innovative technique for the assessment of bone status in young women with anorexia nervosa. Eat Weight Disord 27:3207–3213. 10.1007/s40519-022-01450-2 35896857 10.1007/s40519-022-01450-2 PMC9803747

[37] Caffarelli C, Tomai Pitinca MD, Al Refaie A et al (2022) Ability of radiofrequency echographic multispectrometry to identify osteoporosis status in elderly women with type 2 diabetes. Aging Clin Exp Res 34:121–127. 10.1007/s40520-021-01889-w 34050917 10.1007/s40520-021-01889-w PMC8795029

[38] Valentina Anna D, Maria Luisa B, Greta C et al (2021) First assessment of bone mineral density in healthy pregnant women by means of Radiofrequency echographic multi spectrometry (REMS) technology. Eur J Obstet Gynecol Reprod Biol 263:44–49. 10.1016/J.EJOGRB.2021.06.014 34167032 10.1016/J.EJOGRB.2021.06.014

[39] Lalli P, Mautino C, Busso C et al (2022) Reproducibility and accuracy of the radiofrequency echographic multi-spectrometry for femoral mineral density estimation and discriminative power of the femoral fragility score in patients with primary and disuse-related osteoporosis. J Clin Med. 10.3390/jcm11133761 35807046 10.3390/jcm11133761 PMC9267756

[40] Amorim DMR, Sakane EN, Maeda SS, Sergio Setsuo C (2021) New technology REMS for bone evaluation compared to DXA in adult women for the osteoporosis diagnosis: a real-life experience. Arch Osteoporos 16:175. 10.1007/s11657-021-00990-x 34786596 10.1007/s11657-021-00990-x

[41] Holmes CJ, Racette SB (2021) The utility of body composition assessment in nutrition and clinical practice: an overview of current methodology. Nutrients 13:2493. 10.3390/nu13082493 34444653 10.3390/nu13082493 PMC8399582

[42] Campa F, Toselli S, Mazzilli M et al (2021) Assessment of body composition in athletes: a narrative review of available methods with special reference to quantitative and qualitative bioimpedance analysis. Nutrients 13:1620. 10.3390/nu13051620 34065984 10.3390/nu13051620 PMC8150618

[43] Sam S (2018) Differential effect of subcutaneous abdominal and visceral adipose tissue on cardiometabolic risk. Horm Mol Biol Clin Investig. 10.1515/hmbci-2018-0014 29522417 10.1515/hmbci-2018-0014

[44] Beaudart C, Zaaria M, Pasleau F et al (2017) Health Outcomes of Sarcopenia: A Systematic Review and Meta-Analysis. PLoS ONE 12:e0169548. 10.1371/journal.pone.0169548 28095426 10.1371/journal.pone.0169548 PMC5240970

[45] Albano D, Messina C, Vitale J, Sconfienza LM (2020) Imaging of sarcopenia: old evidence and new insights. Eur Radiol. 10.1007/s00330-019-06573-2 31834509 10.1007/s00330-019-06573-2

[46] Guglielmi G, Ponti F, Agostini M et al (2016) The role of DXA in sarcopenia. Aging Clin Exp Res 28:1047–1060. 10.1007/s40520-016-0589-3 27256078 10.1007/s40520-016-0589-3

[47] Bazzocchi A, Ponti F, Albisinni U et al (2016) DXA: technical aspects and application. Eur J Radiol 85:1481–1492. 10.1016/J.EJRAD.2016.04.004 27157852 10.1016/J.EJRAD.2016.04.004

[48] Guglielmi G, Bazzocchi A (2020) Body composition imaging. Quant Imaging Med Surg 10:1576–1579. 10.21037/qims-2019-bc-13 32742952 10.21037/qims-2019-bc-13 PMC7378092

[49] Messina C, Albano D, Gitto S et al (2020) Body composition with dual energy X-ray absorptiometry: from basics to new tools. Quant Imaging Med Surg 10:1687–1698 32742961 10.21037/qims.2020.03.02 PMC7378094

[50] Damilakis J, Adams JE, Guglielmi G, Link TM (2010) Radiation exposure in X-ray-based imaging techniques used in osteoporosis. Eur Radiol 20:2707–2714. 10.1007/s00330-010-1845-0 20559834 10.1007/s00330-010-1845-0 PMC2948153

[51] Nana A, Slater GJ, Stewart AD, Burke LM (2015) Methodology review: using dual-energy X-Ray absorptiometry (DXA) for the assessment of body composition in athletes and active people. Int J Sport Nutr Exerc Metab 25:198–215. 10.1123/ijsnem.2013-0228 25029265 10.1123/ijsnem.2013-0228

[52] Kaul S, Rothney MP, Peters DM et al (2012) Dual-energy X-ray absorptiometry for quantification of visceral fat. Obesity (Silver Spring) 20:1313–1318. 10.1038/OBY.2011.393 22282048 10.1038/OBY.2011.393 PMC3361068

[53] Liu P, Ma F, Lou H, Liu Y (2013) The utility of fat mass index vs body mass index and percentage of body fat in the screening of metabolic syndrome. BMC Public Health. 10.1186/1471-2458-13-629 23819808 10.1186/1471-2458-13-629 PMC3703297

[54] Cruz-Jentoft AJ, Bahat G, Bauer J et al (2019) Sarcopenia: revised European consensus on definition and diagnosis. Age Ageing 48:16–31. 10.1093/ageing/afy169 30312372 10.1093/ageing/afy169 PMC6322506

[55] Heymsfield SB, Peterson CM, Thomas DM et al (2016) Why are there race/ethnic differences in adult body mass index-adiposity relationships? A quantitative critical review. Obes Rev 17:262–275. 10.1111/OBR.12358 26663309 10.1111/OBR.12358 PMC4968570

[56] Borga M, West J, Bell JD et al (2018) Advanced body composition assessment: from body mass index to body composition profiling. J Investig Med 66:1–9. 10.1136/jim-2018-000722 29581385 10.1136/jim-2018-000722 PMC5992366

[57] Zhao X, Wang Z, Zhang J et al (2013) Estimation of total body skeletal muscle mass in chinese adults: prediction model by dual-energy X-ray absorptiometry. PLoS ONE 8:e53561. 10.1371/journal.pone.0053561 23308254 10.1371/journal.pone.0053561 PMC3538629

[58] Kirk B, Zanker J, Duque G (2020) Osteosarcopenia: epidemiology, diagnosis, and treatment-facts and numbers. J Cachexia Sarcopenia Muscle 11:609–618. 10.1002/JCSM.12567 32202056 10.1002/JCSM.12567 PMC7296259

[59] Paulin TK, Malmgren L, McGuigan FE, Akesson KE (2024) Osteosarcopenia: prevalence and 10-year fracture and mortality risk—a longitudinal, population-based study of 75-year-old women. Calcif Tissue Int 114:315–325. 10.1007/S00223-023-01181-1 38300303 10.1007/S00223-023-01181-1 PMC10957698

[60] Baim S, Binkley N, Bilezikian JP et al (2008) Official positions of the international society for clinical densitometry and executive summary of the 2007 ISCD position development conference. J Clin Densitom 11:75–91. 10.1016/j.jocd.2007.12.007 18442754 10.1016/j.jocd.2007.12.007

[61] Chuang ML, Massaro JM, Levitzky YS et al (2012) Prevalence and distribution of abdominal aortic calcium by gender and age group in a community-based cohort (from the Framingham Heart Study). Am J Cardiol 110:891–896. 10.1016/j.amjcard.2012.05.020 22727181 10.1016/j.amjcard.2012.05.020 PMC3432173

[62] Yang S-W, Yang H-F, Chen Y-Y, Chen W-L (2021) Unraveling the link between metabolic syndrome and abdominal aortic calcification. Nutr Metab Cardiovasc Dis 31:464–471. 10.1016/j.numecd.2020.10.003 33223398 10.1016/j.numecd.2020.10.003

[63] Criqui MH, Denenberg JO, McClelland RL et al (2014) Abdominal aortic calcium, coronary artery calcium, and cardiovascular morbidity and mortality in the multi-ethnic study of atherosclerosis. Arterioscler Thromb Vasc Biol 34:1574–1579. 10.1161/ATVBAHA.114.303268 24812323 10.1161/ATVBAHA.114.303268 PMC4153597

[64] Sethi A, Taylor DL, Ruby JG et al (2022) Calcification of the abdominal aorta is an under-appreciated cardiovascular disease risk factor in the general population. Front Cardiovasc Med 9:1003246. 10.3389/fcvm.2022.1003246 36277789 10.3389/fcvm.2022.1003246 PMC9582957

[65] Levitzky YS, Cupples LA, Murabito JM et al (2008) Prediction of intermittent claudication, ischemic stroke, and other cardiovascular disease by detection of abdominal aortic calcific deposits by plain lumbar radiographs. Am J Cardiol 101:326–331. 10.1016/j.amjcard.2007.08.032 18237594 10.1016/j.amjcard.2007.08.032

[66] Leow K, Szulc P, Schousboe JT et al (2021) Prognostic value of abdominal aortic calcification: a systematic review and meta-analysis of observational studies. J Am Heart Assoc 10:e017205–e017205. 10.1161/JAHA.120.017205 33439672 10.1161/JAHA.120.017205 PMC7955302

[67] Majjad A, Ghassem MA, Toufik H et al (2020) Relationship between vertebral fracture prevalence and abdominal aortic calcification in women with rheumatoid arthritis. Bone 141:115599. 10.1016/j.bone.2020.115599 32822872 10.1016/j.bone.2020.115599

[68] Wu M, Liu Y, Zhong C et al (2021) Osteoporosis was associated with severe abdominal aortic calcification based on a cross-sectional study. Arch Osteoporos. 10.1007/s11657-021-00927-4 34013479 10.1007/s11657-021-00927-4

[69] Gebre AK, Lewis JR, Leow K et al (2022) Abdominal aortic calcification, bone mineral density, and fractures: a systematic review and meta-analysis of observational studies. J Gerontol Ser A. 10.1093/gerona/glac171 10.1093/gerona/glac171 36000920

[70] Setiawati R, Di CF, Rahardjo P et al (2016) Quantitative assessment of abdominal aortic calcifications using lateral lumbar radiograph, dual-energy X-ray absorptiometry, and quantitative computed tomography of the spine. J Clin Densitom 19:242–249. 10.1016/j.jocd.2015.01.007 25708122 10.1016/j.jocd.2015.01.007

[71] Schousboe JT, Lewis JR, Kiel DP (2017) Abdominal aortic calcification on dual-energy X-ray absorptiometry: Methods of assessment and clinical significance. Bone 104:91–100. 10.1016/j.bone.2017.01.025 28119178 10.1016/j.bone.2017.01.025

[72] Glüer C-C (2017) 30 years of DXA technology innovations. Bone 104:7–12. 10.1016/j.bone.2017.05.020 28552661 10.1016/j.bone.2017.05.020

[73] Kauppila LI, Polak JF, Cupples LA et al (1997) New indices to classify location, severity and progression of calcific lesions in the abdominal aorta: a 25-year follow-up study. Atherosclerosis 132:245–250. 10.1016/s0021-9150(97)00106-8 9242971 10.1016/s0021-9150(97)00106-8

[74] Schousboe JT, Wilson KE, Kiel DP (2006) Detection of abdominal aortic calcification with lateral spine imaging using DXA. J Clin Densitom 9:302–308. 10.1016/j.jocd.2006.05.007 16931348 10.1016/j.jocd.2006.05.007

[75] Schousboe JT, Wilson KE, Hangartner TN (2007) Detection of aortic calcification during vertebral fracture assessment (VFA) compared to digital radiography. PLoS ONE 2:e715–e715. 10.1371/journal.pone.0000715 17684561 10.1371/journal.pone.0000715 PMC1933602

[76] Wilson PWF, Kauppila LI, O’Donnell CJ et al (2001) Abdominal aortic calcific deposits are an important predictor of vascular morbidity and mortality. Circulation 103:1529–1534. 10.1161/01.cir.103.11.1529 11257080 10.1161/01.cir.103.11.1529

[77] Bazzocchi A, Ciccarese F, Diano D et al (2012) Dual-energy X-ray absorptiometry in the evaluation of abdominal aortic calcifications. J Clin Densitom 15:198–204. 10.1016/j.jocd.2011.11.002 22321658 10.1016/j.jocd.2011.11.002

[78] Cecelja M, Frost ML, Spector TD, Chowienczyk P (2013) Abdominal aortic calcification detection using dual-energy X-ray absorptiometry: validation study in healthy women compared to computed tomography. Calcif Tissue Int 92:495–500. 10.1007/s00223-013-9704-z 23407824 10.1007/s00223-013-9704-z

[79] Kelly TL, Wilson KE, Heymsfield SB (2009) Dual energy X-ray absorptiometry body composition reference values from NHANES. PLoS ONE 4:e7038. 10.1371/journal.pone.0007038 19753111 10.1371/journal.pone.0007038 PMC2737140

